# A Tribute to Disorder in the Genome of the Bloom-Forming Freshwater Cyanobacterium *Microcystis aeruginosa*

**DOI:** 10.1371/journal.pone.0070747

**Published:** 2013-08-12

**Authors:** Jean-François Humbert, Valérie Barbe, Amel Latifi, Muriel Gugger, Alexandra Calteau, Therese Coursin, Aurélie Lajus, Vanina Castelli, Sophie Oztas, Gaëlle Samson, Cyrille Longin, Claudine Medigue, Nicole Tandeau de Marsac

**Affiliations:** 1 Unité des Cyanobactéries, Institut Pasteur, Centre National de la Recherche Scientifique Unité de Recherche Associée 2172, Paris, France; 2 Institut de Génomique, Commissariat à l'énergie atomique et aux énergies alternatives-Génoscope, Evry, France; 3 Laboratoire de Chimie Bactérienne, Aix-Marseille Université, Centre National de la Recherche Scientifique Unité Mixte de Recherche 7283, Marseille, France; 4 Laboratoire d'Analyse Bioinformatique en Génomique et Métabolisme, Commissariat à l'énergie atomique et aux énergies alternatives-Génoscope, Centre National de la Recherche Scientifique Unité Mixte de Recherche 8030, Evry, France; Université Claude Bernard - Lyon 1, France

## Abstract

*Microcystis aeruginosa* is one of the most common bloom-forming cyanobacteria in freshwater ecosystems worldwide. This species produces numerous secondary metabolites, including microcystins, which are harmful to human health. We sequenced the genomes of ten strains of *M. aeruginosa* in order to explore the genomic basis of their ability to occupy varied environments and proliferate. Our findings show that *M. aeruginosa* genomes are characterized by having a large open pangenome, and that each genome contains similar proportions of core and flexible genes. By comparing the GC content of each gene to the mean value of the whole genome, we estimated that in each genome, around 11% of the genes seem to result from recent horizontal gene transfer events. Moreover, several large gene clusters resulting from HGT (up to 19 kb) have been found, illustrating the ability of this species to integrate such large DNA molecules. It appeared also that all *M. aeruginosa* displays a large genomic plasticity, which is characterized by a high proportion of repeat sequences and by low synteny values between the strains. Finally, we identified 13 secondary metabolite gene clusters, including three new putative clusters. When comparing the genomes of *Microcystis* and *Prochlorococcus*, one of the dominant picocyanobacteria living in marine ecosystems, our findings show that they are characterized by having almost opposite evolutionary strategies, both of which have led to ecological success in their respective environments.

## Introduction

Cyanobacteria play an important role in aquatic ecosystems (e.g. [1], [2]) but they can also disrupt the functioning of these ecosystems and their use by humans, because of the ability of several species to proliferate and to produce harmful toxins (e.g. [3]). In recent years, several studies have shown that sharply contrasting ecological strategies have allowed cyanobacteria to live in a large range of habitats in the euphotic zone of aquatic ecosystems. For example, in *Prochlorococcus marinus*, one of the dominant picocyanobacterial genera in marine ecosystems, different ecotypes occupy various ecological niches mainly defined by the availability of nutrients and light [4]–[7]. In contrast, in *Microcystis aeruginosa*, one of the most common toxic bloom-forming species in eutrophic freshwater ecosystems, no evidence of ecotype differentiation has been found. Moreover, population genetic studies have revealed that *M. aeruginosa* populations are characterized by wide genetic diversity and the lack of biogeographical patterns of genetic differentiation, suggesting that *M. aeruginosa* strains are able to proliferate in a wide range of ecosystems [8], [9].

Hence, one exciting scientific challenge is to elucidate the genomic basis of the different ecological strategies developed by the *Prochlorococcus* and *Microcystis* genera. With this goal, the genomes of many *P. marinus* strains have recently been sequenced (for a review, see [10]). These studies have indicated that almost all sequenced *P. marinus* genomes are characterized by a small size that seems to result from a genome reduction process. However, despite their small size, these genomes have acquired a large number of genes by horizontal gene transfer (HGT) [11]–[15]. Thus, the combination of a niche specialization process involving genome reduction [16], [17], and a high adaptive potential with gene acquisition by HGT, might explain the ecological success of this species.

In contrast to *Prochlorococcus*, only two *M. aeruginosa* strains have been sequenced so far. From these first two studies, *M. aeruginosa* genomes appear to display unusual plasticity, reflected in a large number of repeated sequences and low synteny values between them [18], [19], but these findings should be confirmed on a greater number of strains. In addition, data are needed about the size of the core and pangenome and on the impact of HGT events on the ability of members of this species to colonize various environments. In this goal, we sequenced the genomes of ten other *M. aeruginosa* strains, selected on their phylogenetic relatedness, their geographical origins, and their ability to produce various secondary metabolites.

## Materials and Methods

### *M. aeruginosa* strain culture and DNA isolation

Axenic strains of *M. aeruginosa* were from the Pasteur Culture collection of Cyanobacteria (PCC) and from the NIES collection (T1-4). Cells were grown at 25°C in BG11_0_ supplemented with 2 mM NaNO_3_ and 10 mM NaHCO_3_ [20]. They were harvested by centrifugation (10,000 *g*, 10 min, 18°C), washed twice with sterile distilled water, and kept frozen until DNA extraction.

DNA extraction from the frozen pellets was carried out using Genomic DNA isolation-NucleoBond ® AX (Macherey-Nagel, Hoerdt, France) according to the manufacturer's instructions.

### Sequencing and assembling methods

The sequences of the ten *M. aeruginosa* genomes were obtained by combining several approaches. First, a single read library was constructed for each of the ten *M. aeruginosa* strains and sequenced with the GSflex version (250 nt length), until 16 to 25-fold coverage was obtained. Then, 3 to 8-fold coverage 454 Titanium reads (average 450 nt length), obtained from mate-paired libraries with 2–3 kb or 6–8 kb insert sizes, were added. In agreement with other *de novo* sequencing projects, the percentage of 454 reads used for assembly was approximately 97% and the percentage of Illumina reads used for error correction around 93%. The assembly was performed using Newbler Software (v2.3, Roche). All sequences, which were not included in scaffolds after the assembly process and which displayed a ≥500 bp length, were considered to be scaffolds (see Table S1). In order to improve the quality of the sequences, Illumina reads (51 nt length) were mapped onto all the scaffolds, using SOAP (http://soap.genomics.org.cn) as described by Aury *et al.* [21].

Both coding sequence prediction and automatic annotation were performed by using the Microscope platform, a web-based framework for the systematic and efficient revision of microbial genome annotation (http://www.genoscope.cns.fr/agc/microscope) [22]. Expert validations were carried out for specific genes. This platform was also used for the comparative genome analyses performed on the twelve *Microcystis* genomes and on those of other cyanobacterial species.

The overall characteristics of the ten *M. aeruginosa* genomes are reported in Table S1. Despite high genome coverage values, from 27 to 121 scaffolds were obtained per strain at the end of the assembly. The number of scaffolds was positively correlated to the size of the genomes (Spearman test, P<0.05).

### Phylogenetic analyses

We used two datasets to reconstruct phylogenetic relationships between twelve *M. aeruginosa* strains (the ten newly sequenced ones and two publicly available ones, PCC 7806 and NIES-843): (i) seven housekeeping genes used by Tanabe *et al.* [23], and (ii) 1989 genes from the core genome of the twelve *M. aeruginosa* strains. These 1989 genes among the 2462 core genes correspond to the orthologous genes displaying at least 80% sequence identity over at least 80% of the length of the smallest protein. Before concatenation, the homologous sequences of each gene were aligned using the MUSCLE software with default parameters [24] and the alignments were filtered by using the program GBLOCKS allowing half gap positions [25].

For each dataset, we computed trees using PHYML [26] and we used the Jones–Taylor–Thornton model of amino acid substitution for the protein dataset (core genes), and the Gamma Time Reversible model for the nucleic dataset (housekeeping genes). Heterogeneities between sites were estimated using gamma-distributed rate variation (4 categories), the alpha parameter was computed using PHYML, tree topologies were explored using Nearest Neighbor Interchanges and we used the tlr option to optimise the topology, the branch lengths and rate parameters of the starting trees. One hundred bootstrap replicates were performed to assess the statistical support of each node.

### Bioinformatic analyses

All the bioinformatics analyses were performed by using tools provided by the MicroScope platform [22].

The orthoMCL program (version 1.4), which uses a Markov Cluster algorithm, was used to compute the core and the pangenome of *M. aeruginosa*. Putative orthologies were defined as gene pairs satisfying an alignment threshold of at least 50% amino acid sequence identity over at least 50% of the length of the smallest protein.

The proportion of repeats was estimated using the Repseek algorithm. This algorithm is a fast two-step method (seed detection followed by their extensions), which allows finding large degenerate repeats within or between large DNA sequences [27].

The synteny values representing the percentage of CDSs belonging to a synteny group were estimated by taking into account CDSs sharing at least 35% sequence identity on 80% of the length of the smallest protein, with a gap parameter (number of consecutive genes not involved in synteny), which was set to five.

Finally, the evaluation of horizontal gene transfers was performed (i) by estimating the proportion of genes displaying more than 20% difference in their GC content compared to the mean GC content of the whole genome, and (ii) by the implementation of Interpolated Variable Order Motifs (IVOMs), which exploits compositional biases using variable order motif distributions (2mer to 8mer). This implementation was achieved by using the Alien-Hunter application included in the MicroScope platform.

### Secondary metabolites gene cluster identification

A modified version of the complete genome scanning pipeline for searching secondary metabolites (http://nrps.igs.umaryland.edu/nrps/2metdb/) implemented on the Microscope platform was used to detect NRPS/PKS genes [28]. Each gene within a cluster was compared to its syntenic counterpart at the amino-acid level in the reference genome to obtain the deduced amino-acid sequence identity. The genome of the *M. aeruginosa* strain PCC 7806 [19] was used as reference but when the compared gene cluster was absent from this genome, those of PCC 7941, PCC 9432, PCC 9806 and NIES-843 [18] or the gene cluster psm3 from *Microcystis* sp. K139 [29] were used. Adenylation specificity was checked using online NRPS predictor (http://www-ab.informatik.uni-tuebingen.de/software/NRPSpredictor; [30]).

## Results

### General features of the ten *M. aeruginosa* genomes

The size of the ten new *M. aeruginosa* genomes ranged from 4.2 to 5.2 Mbp. The GC-content value was around 43% for each of the genomes, and the Coding DNA Sequence (CDS) density of ∼81% with approximately one gene per kb. From one to five tRNA genes were found for each of the 20 amino acids in all the *M. aeruginosa* genomes, with differences in the number of copies for tRNA-Thr, tRNA-Ile and tRNA-Met (Table S2). By performing a PCA analysis on the main characteristics of the twelve *M. aeruginosa* genomes described in Table 1 (data not shown), the NIES-843 genome was clearly distinguishable from all the other genomes. Finally, a comparison of the composition of the Minimal Gene Set [31] was performed on all the *M. aeruginosa* genomes with the goal to evaluate the quality of the ten new sequenced ones. Among the 205 genes composing this Minimal Gene Set, none of the genes found in NIES-843 [18] and in PCC7806 [19] was lacking in only one of the ten new genomes (Table S3). A restricted number of them displayed a heterogeneous distribution among the twelve strains, as for example *pgi* (phosphoglucose isomerase), which was found in two copies in the two strains belonging to the sub-clade 1 (see bellow), and only in one copy in the other ones.

**Table 1:** General features of the ten new *Microcystis aeruginosa* genomes and of the two previously available (PCC 7806 and NIES-843).

Strain/Culture collection reference	Location and date isolation	Genome size (Mbp)	Status	SC number	GC%	CDS number	Average CDS length (bp)	IG length (bp)	Coding density	rDNA clusters number	Average gene length (bp)	Ref
**PCC 7941**	Little Rideau Lake, CAN (1954)	4.8	WGS: CAIK00000000–CAIK01000372	77	42.6	4669	839	227	82.1	**1**	1019	This work
**PCC9432**	Little Rideau Lake, CAN (1954)	5.0	WGS: CAIH0000000–CAIH01000377	132	42.5	4952	822	235	81.9	**1**	1000	This work
**PCC 9443**	Fish pond, Landjia, RCA (1994)	5.1	WGS: CAIJ00000000–CAIJ01000735	221	42.8	5055	818	274	80.6	**1**	1011	This work
**PCC 9701**	Guerlesquin dam, F (1996)	4.7	WGS: CAIQ01000001–CAIQ01000550	172	42.8	4668	829	251	81.8	**1**	1010	This work
**PCC 9717**	Rochereau dam, F (1996)	5.2	WGS: CAII00000000–CAII01000881	264	42.8	5164	807	295	79.7	**1**	1009	This work
**PCC 9806**	Oskosh, USA (1975)	4.2	WGS: CAIL00000000–CAIL01000236	93	43.1	4123	833	242	80.9	**1**	1026	This work
**PCC 9807**	Hartbeespoort dam, Pretoria, ZA (1973)	5.1	WGS: CAIM00000000–CAIM01000759	267	42.7	5049	811	286	80.3	**1**	1006	This work
**PCC 9808**	Malpas dam, AUS (1973)	5.0	WGS: CAIN00000000–CAIN01000422	141	42.4	5044	811	241	81.4	**1**	992	This work
**PCC 9809**	Lake Michigan, USA (1982)	4.9	WGS: CAIO0000000–CAIO01000789	303	42.8	4990	806	279	81.3	**1**	988	This work
**T1-4**	Bangkok, T (NA)	4.7	WGS: CAIP01000001–CAIP01000449	145	42.8	4610	830	251	81.3	**1**	1056	This work
**PCC 7806**	Braakman Reservoir, NL (1972)	5.3	WGS: AM778843–AM778958	116	42.0	5496	775	215	81.5	**1**	966	[19]
**NIES-843**	Lake Kasumigaura, J (1997)	5.8	NC_010296	1	42.3	7199	682	159	83.0	**2**	812	[18]

SC number: Number of scaffolds; GC%: Percentage of G/C bases in the genome; CDS number: Number of Coding DNA Sequence; IG length: Average intergenic length; Ref: Reference.

### Estimation of the core and pangenome in *M. aeruginosa*

**Figure 1:**
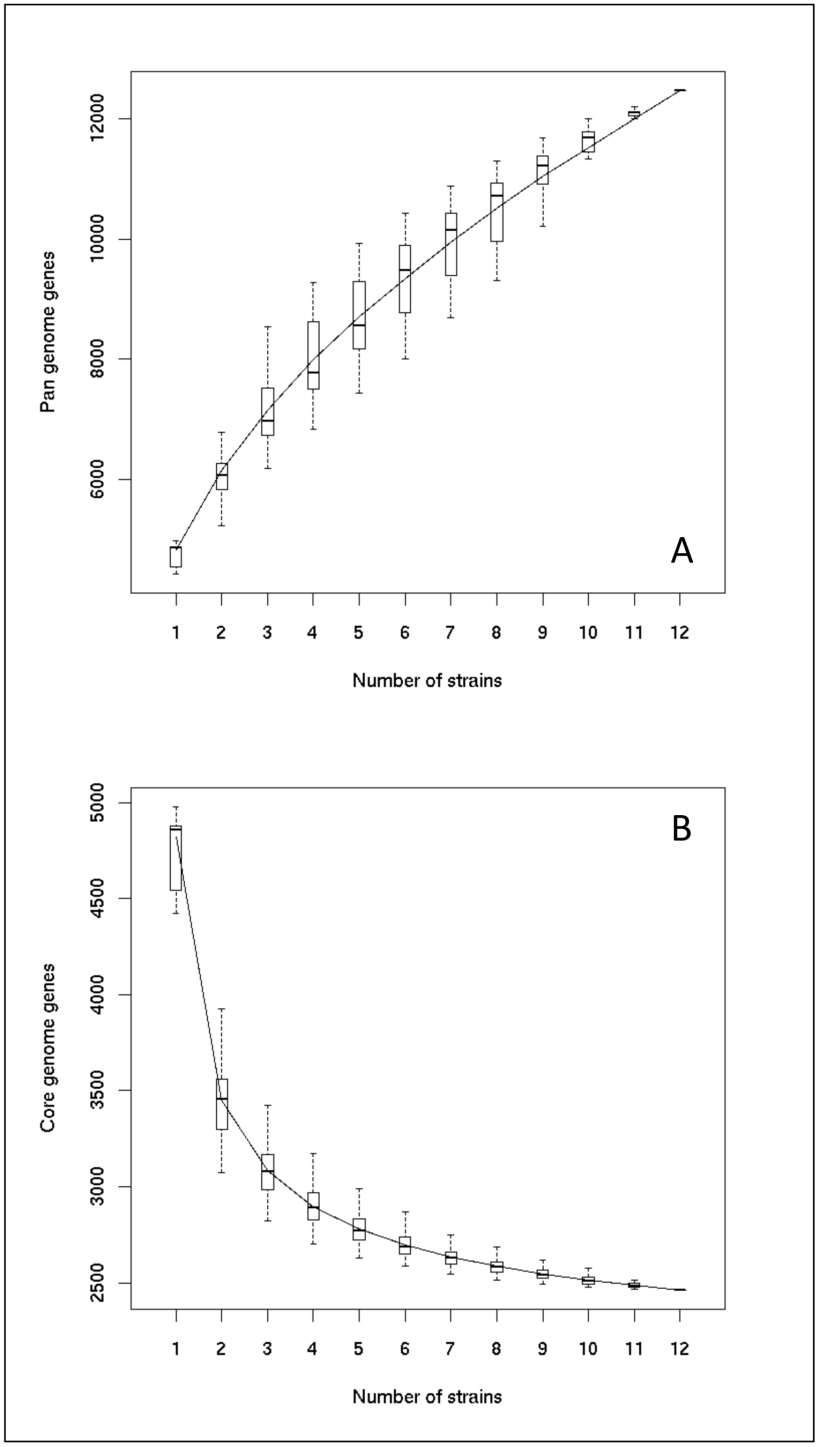
Estimation of the sizes of the pangenome (A) and core genome (B) of *Microcystis aeruginosa* from the twelve *Microcystis aeruginosa* genomes (including the two previously-available genomes of PCC 7806 and NIES-843). The upper and lower edges of the boxes indicate the first quartile and third quartile, respectively, of all different input orders (1000) of the genomes. The central horizontal line indicates the sample median (50th percentile). The central vertical lines extend above and below each box as far as the data extend, to a distance of at most 1.5 times the interquartile range.

As shown in Fig. 1B, the size of the core genome is close to 2462 genes (Table S4). As this curve is not completely asymptotic, the addition of new genomes would probably reduce slightly the size of the *M. aeruginosa* core genome. With regard to the pangenome, the gene accumulation curve does not reach a plateau, indicating that its size (>12000 genes) is largely underestimated (Fig. 1A).

**Figure 2:**
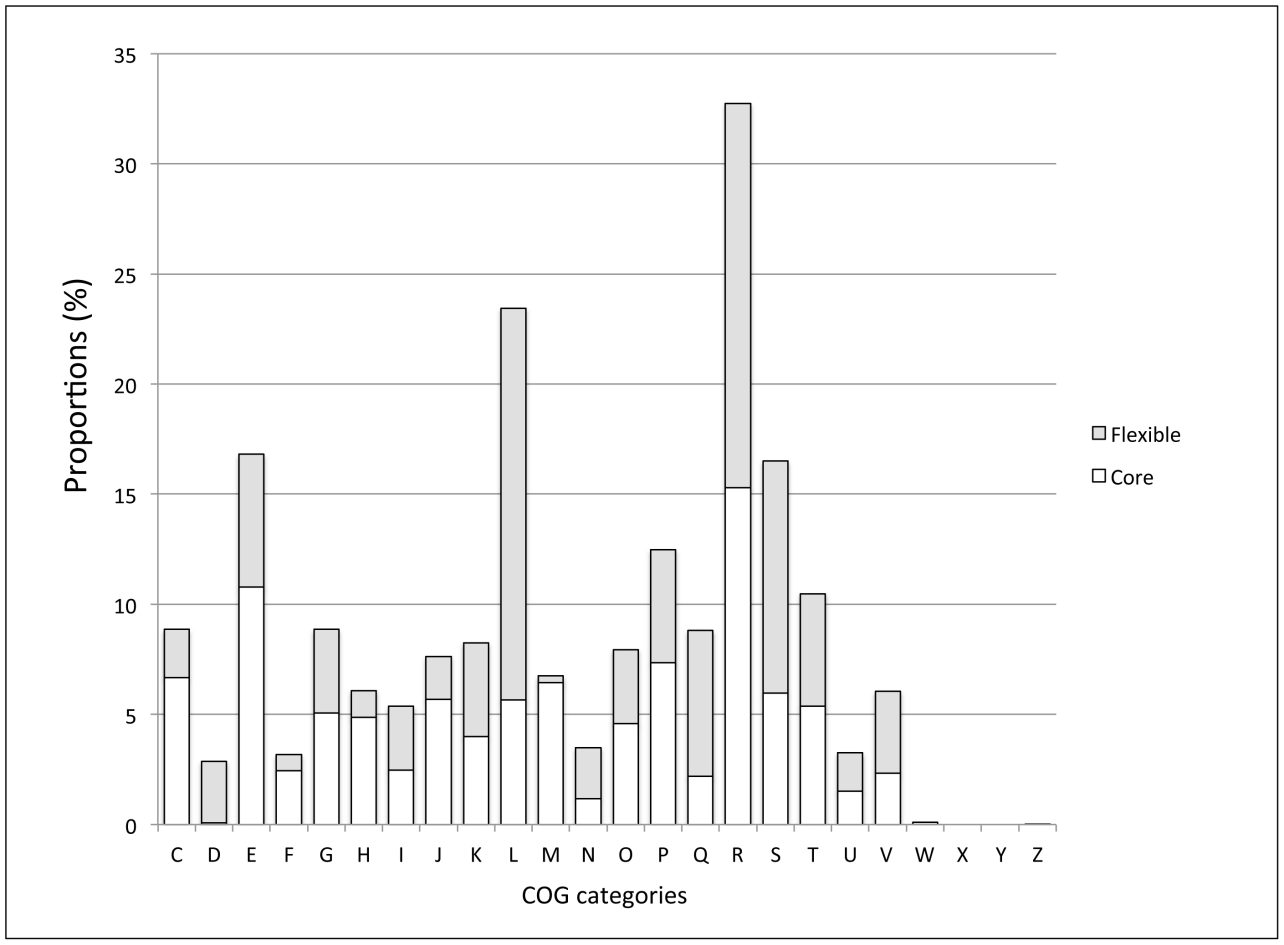
Distribution of the core and flexible genes from all the *Microcystis* genomes in the Clusters of Orthologous Groups (COGs). Only the COG categories containing >1% of the genes in at least one of the two core genomes, are shown in the figure. The functional classifications of the COGs are: **Cellular process and signaling:** (D) Cell cycle control, cell division, chromosome partitioning; (M) Cell wall/membrane/envelope biogenesis; (N) Cell motility (O) Post-translational modification, protein turnover, and chaperones; (T) Signal transduction mechanisms; (U) Intracellular trafficking, secretion, and vesicular transport; (V) Defense mechanisms; (W) Extracellular structures; (Y) Nuclear structure; (Z) Cytoskeleton. **Information storage and processing:** (J) Translation, ribosomal structure and biogenesis; (K) Transcription; (L) Replication, recombination and repair. **Metabolism:** (C) Energy production and conversion; (E) Amino acid transport and metabolism; (F) Nucleotide transport and metabolism; (G) Carbohydrate transport and metabolism; (H) Coenzyme transport and metabolism; (I) Lipid transport and metabolism; (P) Inorganic ion transport and metabolism; (Q) Secondary metabolites biosynthesis, transport, and catabolism. **Poorly characterized:** (R) General function prediction only; (S) Function unknown.

When looking at distribution of the core and flexible genes classified among the Clusters of Orthologous Groups (COGs) categories, it appeared that a smaller proportion of flexible genes was found in C (Energy production and conversion), F (Nucleotide transport and metabolism), H (Coenzyme metabolism), J (Translation, ribosomal structure and biogenesis) and M (Cell envelope biogenesis, outer membrane) categories, compared to that of core genes. In contrast, this proportion of flexible genes is high in the L category (DNA replication, recombination and repair) due to the presence of numerous transposase encoding genes, and also in R (General function prediction only) and S (Unknown function) categories (Fig. 2; Table S4).

### Phylogenetic studies of the twelve *M. aeruginosa* strains and relationships with their geographical origin and their genome size

**Figure 3:**
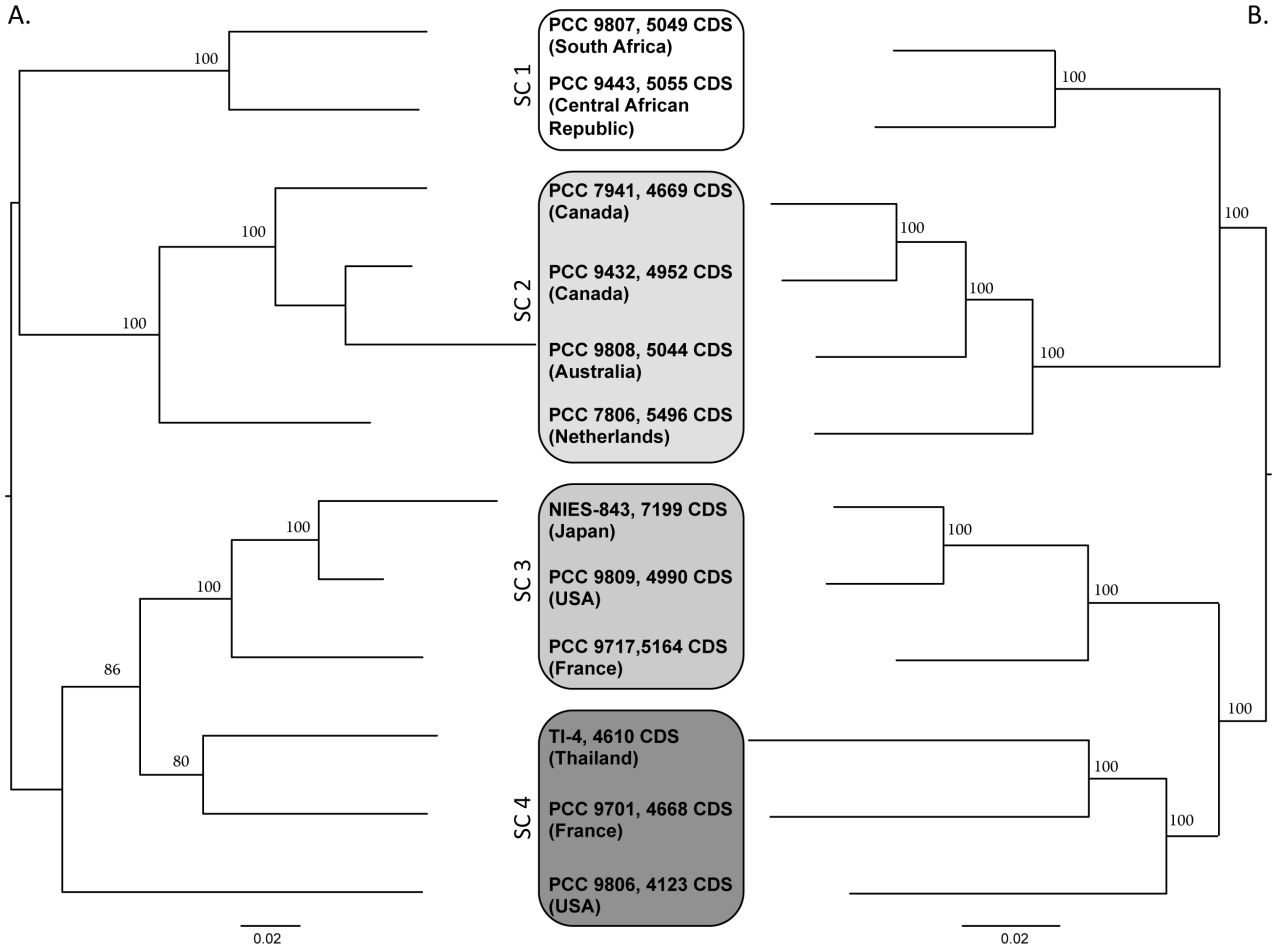
Phylogenetic relationships (Maximum likelihood method) between the twelve *Microcystis aeruginosa* genomes (including the two previously-available genomes of PCC 7806 and NIES-843). A. Phylogeny based on the alignment of seven housekeeping genes (*ftsZ*, *glnA*, *gltX*, *gyrB*, *pgi*, *recA* and *tpi*; 217 informative sites). B. Phylogeny based on the alignment of 1989 genes belonging to the core genome (SC = Subclade ; 144276 informative sites).

A phylogenetic analysis was performed on seven housekeeping genes (*ftsZ*, *glnA*, *gltX*, *gyrB*, *pgi*, *recA* and *tpi*) and on 1989 genes belonging to the core genome. The two trees were congruent, but phylogenetic analysis based on the core genome enhanced the differentiation of subclades (SC) 3 and 4 (Fig. 3). These two trees were also compared to the tree based on the sequence of the 16S–23S rDNA Internal Transcribed Spacer (ITS) (Figure S1). Once again, good overall congruence was found between all these trees, apart from the PCC 9717 strain, which moved from SC3 to SC1 in the ITS tree. Regarding the geographical origin of the strains, it appeared that three subclades (SC2–SC4) contained strains isolated from various continents, suggesting a worldwide distribution of these strains. In contrast, SC1 only contained the two African strains, suggesting a possibly more limited distribution of this subclade.

Finally, to determine whether there was any relationship between the phylogenetic position of the strains and the size of their genome, we performed a Kruskal-Wallis analysis in which we compared the four subclades with regard to the number of CDS in the genome of each strain contained in these subclades. A statistical difference was found at a 7% level, suggesting that a putative link between the phylogenetic position of the strains and the size of their genome might have been found if a larger sample of strains in each of the four subclades had been available.

### Proportion of repeated sequences

**Figure 4:**
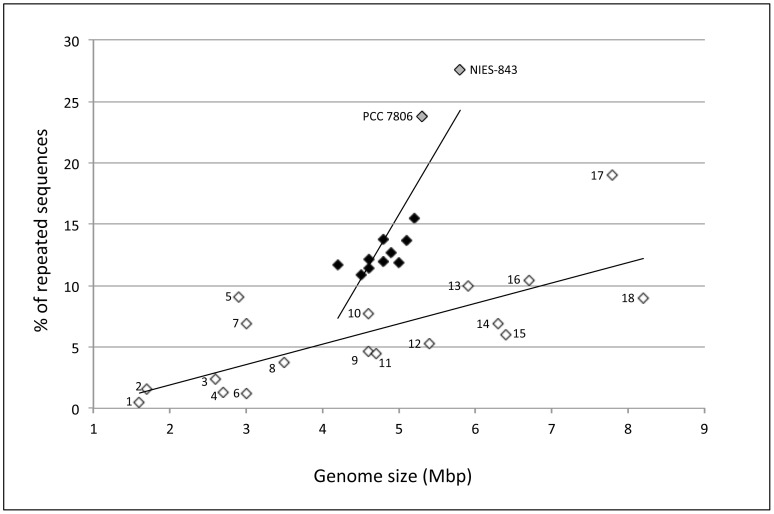
Proportions of repeated sequences according to the size of the genomes in the ten new *Microcystis aeruginosa* genomes (black diamonds), the two previously-available genomes (PCC 7806 and NIES-843) (gray diamonds) and other cyanobacterial genomes (*Prochlorococcus* AS9601 (number 1 in the figure) & MIT 9301 (2); *Oscillatoria* PCC 6506 (16); *Nostoc* ATCC 29133/PCC 73102 (18) & PCC 7120 (15); *Synechococcus* PCC 6301 (4), JA-2-3B (7); JA-3-3ab (5); PCC 7002 (5) & CC 9311 (3); *Synechocystis* PCC 6803 (8), *Trichodesmium* IMS 101 (17); *Anabaena* ATCC 29413 (14); *Cyanothece* PCC 7424 (13), PCC 7425 (12), PCC 8801 (9) & PCC 8802 (11); *Gloeobacter* PCC 7421 (10); white diamonds).

The proportion of repeats (see M&M) was carried out on the twelve *M. aeruginosa* genomes plus a selection of 18 cyanobacterial genomes available in our database. From this analysis, it appears that all the *M. aeruginosa* genomes displayed a higher proportion of repeated sequences than the other cyanobacterial genomes selected for this comparison (Fig. 4). Moreover, the slope of the regression line between the size of the genomes and the proportion of their repeats was much steeper for *M. aeruginosa* genomes than for the others, with NIES-843 and PCC 7806 displaying the highest proportions of repeats.

We also estimated the number of replicated CDS in the twelve *Microcystis* genomes by taking into account two amino-acid sequence similarity levels (70 and 90%). A highly significant relationship was found between the number of CDS they contained and the number of repeated CDS (r^2^_Pearson_ = 0.87, p<0.0001 at 70% sequence similarity; r^2^_Pearson_ = 0.82, p<0.0001 at 90% sequence similarity level).

### Synteny analysis

The synteny values between the twelve *M. aeruginosa* genomes range from 67% to 86% (mean value 76±4%). In order to find out whether the synteny values estimated between the twelve genomes match with the phylogenetic relationships of the corresponding strains, we performed a non-metric MDS analysis on the matrix of these values (Fig. S2). From this figure, it appears that the strains belonging to SC1 and SC2 are clearly distinguished from those belonging to SC3 and SC4. Interestingly, the three strains belonging to SC4, which form the longest branches of the phylogenetic trees (Fig. 3), are the ones most widely scattered in the MDS scatterplot. We could conclude that the phylogenetic diversification of *M. aeruginosa* was accompanied by chromosome rearrangements leading to a rapid decrease in synteny.

As expected, the largest synteny group shared by the twelve *M. aeruginosa* genomes contains genes encoding ribosomal proteins. It is worth noting here that the second largest group comprises eleven genes involved in the transport of various nutrients, such as the gene clusters *cmpABCD* and *nrtABCD* encoding the ABC transporter complex for bicarbonate and that for nitrate, respectively, as well as several genes (including *pstS*, *pstB*, *pstC*) involved in the transport of phosphate.

### Evaluation of horizontal gene transfer

**Figure 5:**
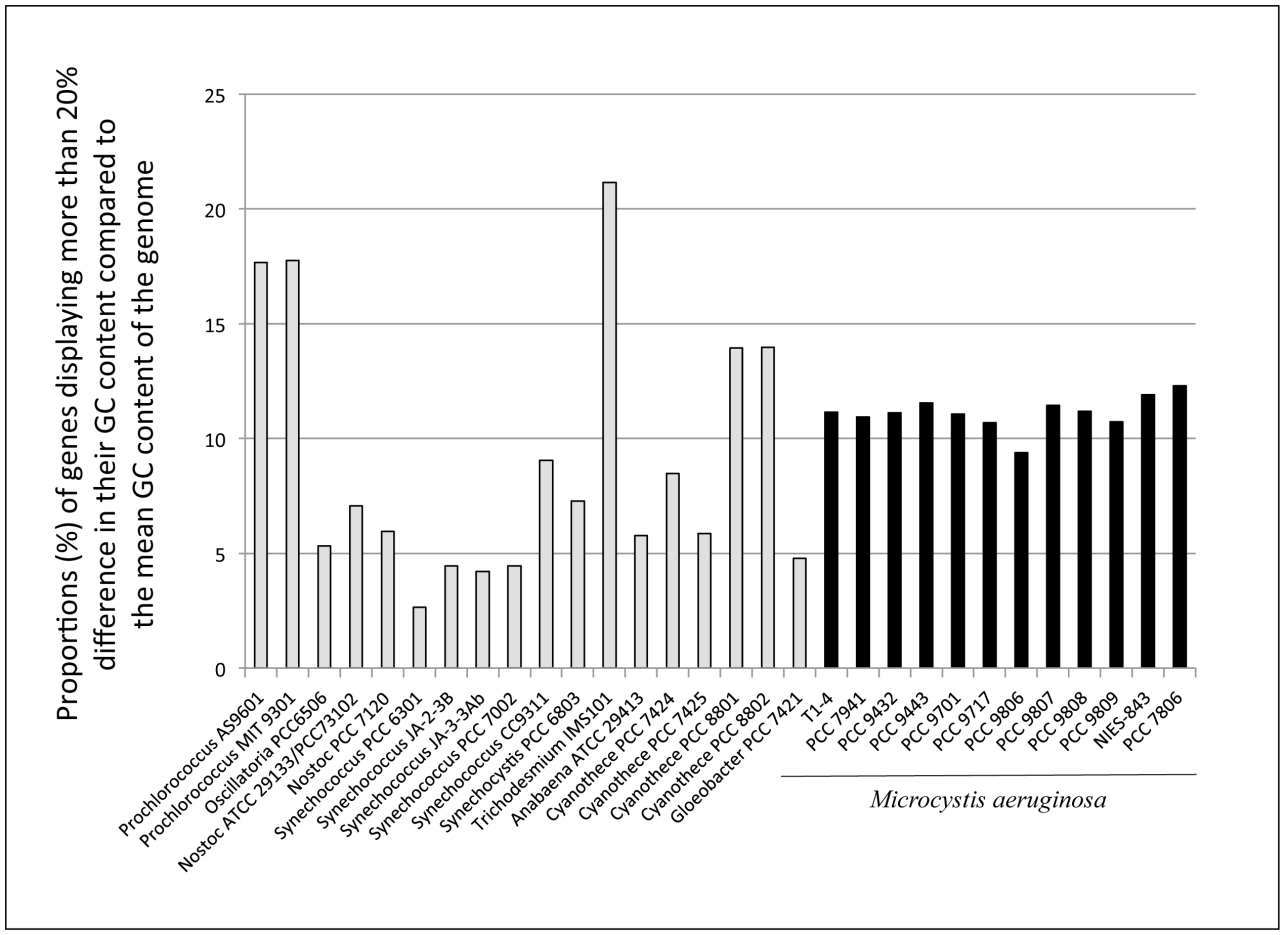
Proportions of genes displaying a ±20% difference in their GC content, compared to the mean GC content of their whole genome in the twelve *Microcystis aeruginosa* genomes (including the two previously-available genomes of PCC 7806 and NIES-843) and in other cyanobacterial genomes.

All the *Microcystis* genomes contained around 11% of genes displaying more than 20% difference in their GC content compared to the mean GC content of the whole genome (Fig. 5; Table S5). This proportion is intermediate between the high percentage (>17%) of these genes found in the genomes of *Prochlorococcus marinus* (AS9601, MIT 9301) and *Trichodesmium erythraeum* (IMS101), and the lowest percentage (<3%) found in *Synechococcus elongatus* PCC 6301.

From 106 to 247 IVOMs were found in the *M. aeruginosa* genomes. No relationship was found between the number of IVOMs and the size of the genome (or the number of CDSs) when we considered only the ten new genomes and the PCC 7806 strain. However, a positive correlation was found when the NIES-843 genome was included in the analysis. This correlation might be due to the fact that the NIES-843 genome is larger and contains a huge number (247) of IVOMs.

Parallel to the analysis of horizontal transfer of genetic information, we looked for genes that are strain-specific among the twelve *M. aeruginosa* genomes. From 100 to 225 strain-specific genes were found in eleven of the *M. aeruginosa* genomes, and as many as 805 in NIES-843. Less than 10% of these strain-specific genes displayed over 40% identity between their deduced amino acid sequences and sequences from the Swissprot database.

Among them, we found for example, in PCC 7806, a strain-specific cluster (IPF 1564–1566) containing three genes involved in the biosynthesis of sucrose (*sppA*, *susA* and *spsA*). These three genes displayed a 40–70% amino-acid sequence identity with sequences found in various cyanobacterial genera (e.g. *Nodularia*, *Nostoc*, *Cyanothece*…). As expected, we found in T1-4, the only strain among the 12 sequenced *Microcystis*, which synthesizes phycoerythrin [32], the genes required for the biosynthesis of this red phycobiliprotein and those involved in the regulation of their expression (data not shown). A 19-kb gene cluster (IPF_1564–1566) encoding for unknown proteins was identified only in the *M. aeruginosa* PCC 9808 genome. This cluster was also found with a similar physical organization and high amino acid sequence identities, in *Lyngbya majuscula* 3L (*Cyanobacteria*; Taxonomy ID: 489825) and *Herpetosiphon aurantiacus* DSM 785 (*Chloroflexi*; Taxonomy ID: 316274) (Fig. S3), and with lower amino acid sequence identities in the genomes of several proteobacteria and of two Archaea. All the genes belonging to these three clusters display a 20% deviation in their GC content, compared to the mean GC content of the genome of the strains in which they have been found.

### Secondary metabolites

**Figure 6:**
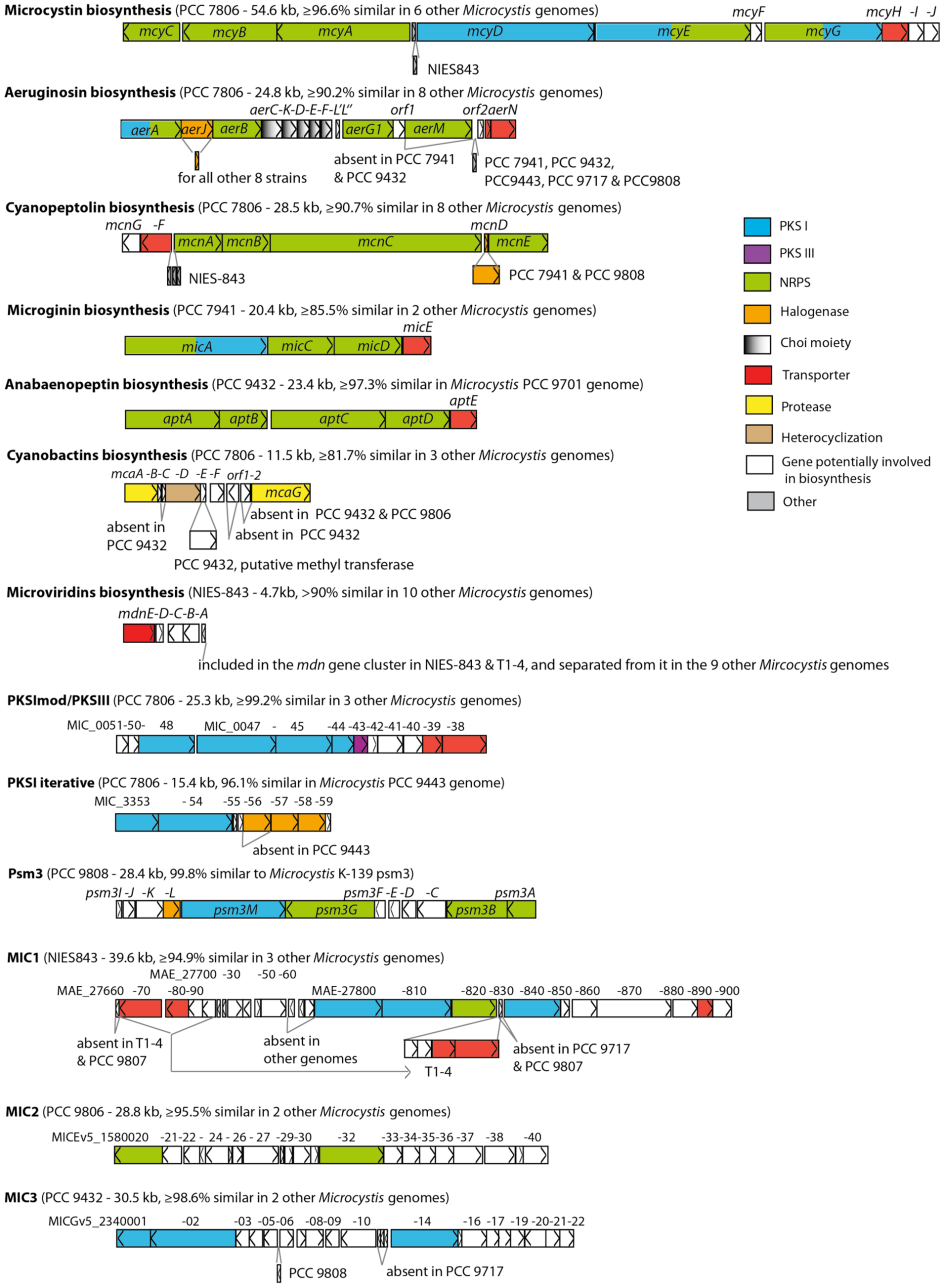
Schematic representation of all the secondary metabolite gene clusters found in the twelve *Microcystis aeruginosa* genomes (including the two previously-available genomes of PCC 7806 and NIES-843). For each biosynthesis cluster, the sketch corresponds to the gene cluster present in the reference strain genome, its size in kb and the amino acid sequence identity estimated for the orthologous region in the other *Microcystis* genomes. The reference strain genome corresponds to the one indicated in Table 2.

Eleven gene clusters encoding non-ribosomal peptide synthetase (NRPS) and/or polyketide synthase (PKS), and two ribosomal ones predicted to be involved in the biosynthesis of secondary metabolites, were found among the *Microcystis* genomes (Table 2). Each genome contained between two and nine such gene clusters accounting for 1.0 to 3.4% of the total genome size. Seven of these clusters encode enzymes for the biosynthesis of known metabolites (microcystins, aeruginosins, cyanopeptolins, microginins, anabaenopeptins, cyanobactins and microviridins), whereas the six remaining clusters encode enzymes for the biosynthesis of so-far unidentified products (Fig. 6).

**Table 2:** Distribution and amino-acid sequence identity of 13 gene clusters involved in the biosynthesis of secondary metabolites in the twelve *Microcystis* genomes (including the two previously available, PCC 7806 and NIES-843).

	Gene cluster		*mcy*	*aer*	*mcn*	*mic*	*apt*	*mca*	*mdn*	PKSImod/PKSIII	PKSI iterat	*psm3*	MIC1	MIC2	MIC3
	Size (kb)		51.8–54.6	12.9–24.8	21.8–30.2	19.6–20.5	23.4	10.7–11.5	4.7	25.3	15.2–16.1	28.4	37.2–39.6	25.8–28.8	30.0–30.6
Strain	% of genome	Nb clust/strain	Aa seq ident (Detection$)	Aa seq ident (Detection$)	Aa seq ident (Detection$)	Aa seq ident	Aa seq ident	Aa seq ident	Aa seq ident for *mdnB-E*	Aa seq ident	Aa seq ident	Aa seq ident to AB279593	Aa seq ident	Aa seq ident	Aa seq ident
**PCC 7941**	3.3	6	98.3 (+)	94.9 (−)	95.3 (+)	**Ref.**	-	-	98.2	99.2	-	-	-	-	-
**PCC 9432**	3.4	9	- (−)	94.8 (+)	91.2 (+)	89.9*	**Ref.**	81.7#	98.2	99.2	partial£	-	-	-	**Ref.**
**PCC 9443**	2.3	4	96.9 (na)	95.5 (+)	*mcnG-F* £ (−)	-	-	-	90.0	-	96.1	-	-	-	-
**PCC 9701**	1.8	4	- (na)	- (na)	90.7* (+)	85.5	97.3	-	98.5	-	-	-	-	-	-
**PCC 9717**	2.8	6	*mcyA-C* £ (na)	93.4 (na)	91.3* (+)	-	-	-	98.1	-	-	-	98.2	95.5*	98.6*
**PCC 9806**	1.0	2	- (na)	- (na)	- (na)	-	-	91.5	*mdnB, mdnD* £	-	-	-	-	**Ref.**	-
**PCC 9807**	2.9	5	97.6 (+)	90.2 (+)	96.1(+)	-	-	-	96.2	-	-	-	96.3	-	-
**PCC 9808**	3.2	6	96.7 (−)	93.2 (−)	92.3 (+)	-	-	-	98.1	-	-	99.8	-	-	99.7
**PCC 9809**	2.9	6	96.9 (+)	93.5 (+)	92.0*(+)	-	-	95.6	98.6	99.3	-	-	-	-	-
**T1-4**	1.6	3	- (na)	- (na)	- (na)	-	-	-	97.1	-	-	-	94.9	96.2	-
**PCC 7806**	3.1	7	**Ref.** (+)	**Ref.** (+)	**Ref.** (+)	-	-	**Ref.**	96.6	**Ref.**	**Ref.**	-	-	-	-
**NIES-843**	2.5	5	96.6 (+)	93.8 (na)	91.6 (na)	-	-	-	**Ref.**	-	-	-	**Ref.**	-	-
No of strains containing the complete gene cluster			7	9	9	3	2	4	11	4	2	1	4	3	3

*Mcy*: Microcystins; *Aer*: Aeruginosins; *mcn*: Cyanopeptolins; *Mic*: Microginins; *Apt*: Anabaenopeptins; *mca*: Cyanobactins; *mdn*: Microviridins; PKSI iterat: PKSI iterative; % of genome: Proportion of genome; Nb clusters/strain: Number of clusters per strain. Aa seq ident: Amino-acid sequence identity; Ref.: Reference strain used for the estimation of the sequence identity.

£ : incomplete gene cluster;

* : on adjacent contigs highly fragmented genome or region;

$ : metabolite detected in previous studies ([58]; Welker, unpublished);

# : probably encodes for another cyanobactin; **(+)**: positive detection of the metabolite in the strain; **(−)**: negative detection of the metabolite in the strain; **na**: not analyzed.

Four of these gene clusters (cyanopeptolins, aeruginosins, microcystin and microviridins) were found in most of the genomes (Table 2), and their deduced amino acid sequences displayed high percentages of amino-acid sequence identity (AASI) (>90%). However, some variability was found in the *aer* gene cluster, both in its composition (presence/absence of *aerJ* and *aerM* for instance) and in the length of the gene sequences (1578 to 4287 bp long for *aerA* in PCC 9432 and PCC 9807). With regard to the *mdn* gene cluster, the MdnB-E proteins were highly conserved (AASI>90%), whereas MdnA, which is involved in precursor synthesis, was less conserved (>52%). Although some *mdn* genes were lost from the PCC 9806 genome, the presence of *mdnB* and *mdnD* in this genome indicated that the microviridin gene cluster was originally present in all the *Microcystis* genomes examined.

All the other gene clusters potentially involved in the synthesis of secondary metabolites were found in only one to four genomes. For example, the proteins involved in the biosynthesis of anabaenopeptins were extremely well conserved (AASI>97%) in PCC 9432 and PCC 9701. On the other hand, the proteins involved in the biosynthesis of microginin and of the cyanobactins (such as microcyclamides) appeared to be less conserved (AASI>85% and >82%, respectively). Similarly, the cyanobactin gene cluster of PCC 9432 displayed considerable differences from that present in PCC 7806.

In addition to these already well-known gene clusters, six orphan gene clusters were also retrieved (Table 2, Fig. 6). The extremities of these gene clusters have been defined by using the available data from the first genome in which they have been found (e.g. PKSImod/PKSIII, PKSI iterative in Frangeul *et al.* [19]). For example, the high synteny and deduced amino acid sequence identity (cluster of 12 adjacent genes with AASI>99.2%) found between the PKSImodular/PKSIII gene clusters in four *M. aeruginosa* genomes revealed that this cluster contained at its 3′end, some additional genes to the ones previously described for this cluster [19] (Fig. 6).

Three new putative NRPS and/or PKS gene clusters (MIC1, MIC2 and MIC3) were retrieved from at least three genomes with high AASI (>92%) and may be involved in the production of so-far unknown compounds. MIC1 is the most promising gene cluster, with adjacent NRPS and PKS genes and RND superfamily efflux transporters. MIC2 and MIC3 were organized as individual thiotemplate modular systems surrounded by putative tailoring enzymes.

Finally, a Correspondence Analysis performed on the distribution of all these gene clusters among the twelve *M. aeruginosa* genomes revealed that three (SC1, SC2 and SC3) of the four subclades defined by our phylogenetic approach cannot be distinguished by their content in these genes (Fig. S4). On the other hand, the three genomes belonging to SC4 are clearly differentiated from the other genomes by the absence of *mcy* and *aer* gene clusters and more generally by their low content of genes involved in the biosynthesis of secondary metabolites.

## Discussion

Despite the multiple sequencing strategies used in this study and the very high sequence coverage, the final assembly process was impeded by the very high proportions of repeated sequences present in the ten new *M. aeruginosa* genomes. However, several observations indicate that a comparative genomic analysis could be undertaken with good confidence. First, the percentage of 454 reads (around 97%) used for assembly was in the same range than that obtained for finished genomes. Second, no gene was lacking in the Minimal Gene Set of the ten new genomes, when compared with that of the *M. aeruginosa* NIES-843 complete genome. Third, an almost asymptotic curve for the core genome was reached, a result that could not be obtained if numerous genes had been missing in these genomes.

The adaptive capacities of *M. aeruginosa*, which may explain its worldwide distribution and its ability to proliferate and to dominate the phytoplankton communities in eutrophic freshwater ecosystems, seem to rely on a particular genome evolutionary strategy. This strategy combines a large genome plasticity, characterized by a high number of repeated sequences, numerous rearrangements and an ability to include new adaptive genes by horizontal gene transfer.

Compared to other cyanobacterial genomes the *M. aeruginosa* genomes display both high proportions of repeated sequences and wide variations in their proportions depending on the strains. The proportions of these repeats appear to be lower in the ten new *M. aeruginosa* genomes than in the two previously-known ones obtained by a Sanger-sequencing approach. The Newbler assembler used for the assembly of the ten new genomes is known to span a lot of repeats from the contigs [33], and so it is likely that we have underestimated the proportions of repeats in the ten new genomes. As previously described by Larsson *et al.* [34], who compared the genomes of different cyanobacterial genera, we also found a positive correlation between the genome size and the number of duplicated genes. It has been hypothesized that a combination of both gene duplication and transfer of orthologous alien genes, without replacement of the native genes, led to an increase in genetic redundancy that contributes to the robustness of biological systems, *i.e.* their ability to continue to function despite external and internal perturbations (e.g. [35]). *A contrario*, a loss of genetic redundancy in bacteria displaying a reductive genome evolution, as observed in numerous pathogens [36] and in oceanic picocyanobacteria of the genus *Prochlorococcus* [15], would result in weak selection for robustness, reflecting the fact that these microorganisms live in stable environments. Thus, the unusually high genetic redundancy in the *M. aeruginosa* genomes might be regarded as an evolutionary strategy that allows this cyanobacterial species to occupy changing environments, such as freshwater ecosystems.

Another peculiar trait of *M. aeruginosa* concerns the low synteny values found for the twelve genomes analyzed in the present study. These low synteny values contrast with the very high 16S rRNA sequence similarities (>99.5%) found between *M. aeruginosa* genomes, and indicate a rapid evolution of the gene organization that probably permits to generate new combinations of genes allowing different adaptive capacities to emerge.

The third feature of the *M. aeruginosa* genomes is that *M. aeruginosa*, like *Prochlorococcus* [14], [37], displays an open pangenome that allows it to acquire new genes. Among them, it was very interesting for example to find the gene cluster involved in the biosynthesis of sucrose in *M. aeruginosa* PCC 7806 alone, because sucrose is known to be involved in the salt acclimation mechanism [38]. Indeed, Guljamow *et al.* [39] had identified actin and profilin genes in PCC 7806 genome, which could be involved in the adaptation to high osmotic stress. Thus, our finding reinforces the hypothesis that PCC 7806 cells may have acquired by HGT two gene clusters that have allowed them to survive and develop in their original habitat (Braakman water reservoir, The Netherlands), which is known to have undergone successive changes in the salt concentration during its history [39]. Another interesting finding was the presence in *M. aeruginosa* PCC 9808 of a large gene cluster (19 kb, IPF_1564–1566) potentially resulting from HGT that illustrates the ability of *M. aeruginosa* to integrate large DNA molecules into its chromosome.

The plasticity of the *M. aeruginosa* genome seems to be linked to the ability of members of this species to bloom in a wide range of ecosystems worldwide. However, such a strategy results in disorder in the organization of the genome, and might also be costly for cell functioning. For example, it has been shown that maintaining redundant genes is costly when these genes belong to the core genes (e.g. [40]). With regard to synteny, it is not clearly understood why selective pressures tend to promote the conservation of gene order in bacterial genomes (e.g. [41]). However, the conservation of chromosome organization in numerous species suggests that this process benefits the cell, and is therefore positively selected for, whereas changes in chromosome organization are counter-selected. In the same time, as recalled by Vicente and Mingorance [42], the plasticity of bacterial genomes resulting from the acquisition and loss of DNA fragments, and also from modifications in gene organization, has multiple effects on the transcriptional networks that can lead, for example, to differential regulation of the same gene in different *M. aeruginosa* strains. Thus, a major challenge in the coming years will be to determine the links between the physiological and ecological characteristics of *M. aeruginosa*, and the selection in this species of a genome evolutionary strategy based on high genome plasticity. Moreover, one of the most interesting issues that will have to be tackled will be also to better understand the impact of the chaotic population dynamics (strong biomass oscillations) in *M. aeruginosa* populations (see for example [43], [44]) on the characteristics of the genome of this species. Indeed, Handel and Rozen (2009) [45] have proposed that the dynamics of evolutionary processes may be linked to the interactions between various parameters, including the variations in population size.

Bacteria are able to cope rapidly and efficiently with different kinds of starvation and stress, which implies that the underlying regulatory network can adjust to many different situations. The overall behavior of bacteria is indeed a result of complex connections between global regulators of gene expression. Since the *M. aeruginosa* strains used in this study originated from different environments, a search was performed for the absence/presence of genes coding for pleiotropic regulators known to allow cyanobacterial cells to cope with stress or starvation conditions (NtcA, Fur, AbrB, PerR, Crp for example) (all these data are available on request from the authors). This analysis indicated that, as expected, genes encoding the regulators required to respond to forms of stress that are common in the ecological niches occupied by the strains studied (high light acclimation, oxidative stress response…) are well conserved in all twelve *M. aeruginosa* genomes. However, specific regulatory networks may optimize the development of a given strain in its ecological niche. These twelve *M. aeruginosa* strains therefore provide an excellent group of very closely related microorganisms for studying the impact of the environment on the plasticity and evolution of regulatory networks.

Genome-wide analyses have recently become very useful tools for finding gene clusters potentially involved in the synthesis of secondary metabolites in several cyanobacterial strains (e.g. [46], [47]). In *M. aeruginosa*, a cyanobacterial species that includes a high number of toxic strains [48]–[50], large variations were found in the organization of some of these clusters, and in their sequences. For example, the organization and size of the *aer* synthetase gene cluster showed more plasticity than previously revealed by Ishida *et al.* [51]. With the exception of strains NIES-843 and T1-4, the *mdn* precursor coding genes were clearly located outside the *mdn* gene cluster in all the other genomes, which corroborates the versatility of the biosynthetic enzyme MdnA shown by Ziemert *et al.* [52]. Interestingly, the cyanobactin gene cluster seems to encode microcyclamide in PCC 7806 [53], [54], and probably in PCC 9806 and 9809, but another type of cyanobactin in PCC 9432. Considering the diversity of the cyanobactins in cyanobacteria [55], *Microcystis* might produce more variants of microcyclamides or perhaps of some other cyanobactins.

Among the 13 gene clusters involved in the biosynthesis of secondary metabolites, microcystins, aeruginosins, cyanopeptolins and microviridins were those most widely distributed in the twelve *Microcystis* genomes, but it appeared that one subclade (SC4) was clearly distinguishable from the others. These three strains displayed the longest branches in the phylogenetic tree and originated from different continents, suggesting that these widely distributed NRPS/PKS gene clusters were lost early in the evolutionary radiation of these strains. The common distribution of the microcystin, aeruginosin and cyanopeptolin metabolites in three other subclades compared to the more sporadic distribution of the other secondary metabolites, suggest that putative interactions lead to their joint conservation. The observation that their gene expression profiles during a day/night cycle are similar [56], is consistent with the above hypothesis.

In conclusion, it seems interesting to compare the main features of the *M. aeruginosa* genomes with those of *P. marinus*, because members of these two cyanobacterial species are characterized by having wide geographical distributions, and play major roles in their respective environments (freswater *versus* oceanic ecosystems). From this comparison it appears that members of both species display very different evolutionary strategies even if some common features can be found in their genome organization. For example, all these genomes contain almost the same proportions of core and flexible genes, even if the mean size of the *M. aeruginosa* genomes exceeds more than two fold that of the *P. marinus* genomes. In the same way, both species display an open pangenome that permits them to colonize various ecological niches.

But in contrast with *Prochlorococcus*, *M. aeruginosa* does not seem to be organized in ecotypes, and its large genome contains a large number of repeat sequences and a high proportion of transposases. These characteristics might permit rapid variation in gene content and the occurrence of new gene combinations allowing *M. aeruginosa* populations to cope with various environmental conditions encountered by this species. Moreover, the *M. aeruginosa* genomes harbor wide genetic diversity with ribosomal and non-ribosomal gene clusters dedicated to synthesis of these bioactive compounds whereas the reduced genomes of *P. marinus* are nearly devoid of all these secondary metabolite biosynthesis gene clusters, even if it has been described that some *Prochlorococcus* strains are able to produce various lantipeptides by using a single biosynthetic enzyme gene [57]. Finally, it is interesting to consider the sharply contrasting evolutionary strategies adopted by *M. aeruginosa* and *P. marinus*, and their ability to dominate their respective habitats. Freshwater ecosystems are very small in comparison to oceanic areas, and consequently are more likely to display rapid fluctuations in their physico-chemical and biological characteristics. Knowing that the differentiation of ecotypes is linked to the occupation of a stable niche, *M. aeruginosa* has developed another winning genomic strategy based on the high plasticity of its genome, to enable it to cope with and colonize unstable freshwater ecosystems efficiently.

## Supporting Information

Table S1 **Overall technical characteristics of the ten new** ***Microcystis aeruginosa*** **genomes.**
(DOCX)

Table S2 **Distribution of the tRNA genes in the twelve** ***Microcystis aeruginosa*** **genomes (including the two previously-available genomes of PCC 7806 and NIES-843).**
(DOCX)

Table S3 **Distribution of the 205 genes from the Minimal Gene Set** [**30**] **in the twelve** ***Microcystis aeruginosa*** **genomes (including the two previously-available genomes of PCC 7806 and NIES-843).**
(XLSX)

Table S4 **Automatic annotation and COG assignation of the** ***Microcystis aeruginosa*** **core genes.**
(XLS)

Table S5 **List of the genes, for the twelve** ***Microcystis*** **genomes, displaying more than 20% difference in their GC content compared to the mean GC content of the whole genome.**
(XLSX)

Figure S1 **Phylogenetic relationships (Neighbor-joining method) between the twelve** ***Microcystis aeruginosa*** **genomes (including the two previously-available genomes of PCC 7806 and NIES-843) based on the alignment of the 16S–23S rDNA Internal Transcribed Spacer.**
(TIF)

Figure S2 **Non-metric MDS analysis performed on the matrix of synteny values estimated between the twelve** ***Microcystis aeruginosa*** **genomes (including the two previously-available genomes of PCC 7806 and NIES-843).** SC1, 2, 3 & 4: Subclades have been defined in the phylogenetic tree based on the core genome (see Fig. 3)
(TIF)

Figure S3 **Comparative gene organization of a strain specific gene cluster found in** ***Microcystis aeruginosa*** **PCC 9808 (MICG_2110008- MICG_2120011) with that found in** ***Lyngbya majuscula*** **and** ***Herpetosiphon auriantiacus*** **genomes.**
(TIF)

Figure S4 **Correspondence analysis performed on the distribution (coded as 1 when all the genes of the cluster were present in a strain; 0.5 when the cluster was not complete and 0 when no gene of the cluster was present) of the clusters of genes involved in the biosynthesis of secondary metabolites among the twelve** ***M. aeruginosa*** **strains (including the two previously-available genomes of PCC 7806 and NIES-843).** SC1, 2, 3 & 4: Sub-clades defined in the phylogenetic tree based on the core genome (see Fig. 3).
(TIF)
