# Supplementary material for: A Tribute to Disorder in the Genome of the Bloom-Forming Freshwater Cyanobacterium Microcystis aeruginosa
Source: PLoS One. 2013 Aug 12;8(8):e70747. doi: 10.1371/journal.pone.0070747 (PMC3741299; doi:10.1371/journal.pone.0070747)
Supplement: Table S1 — Overall technical characteristics of the ten new Microcystis aeruginosa genomes. (DOCX) [file pone.0070747.s001.docx]

**Supplemental Table 1**

| **Strain** | **Genome size (Mbp)** | **454 SR**  **number** | **Cov. 454 SR** | **454 MPr**  **number** | **Cov. 454 MPr** | **MP FS (kb)** | **GAIIX SR**  **number** | **Cov. GAIIX SR** | **Contigs**  **number ≥500nt** | **Number of SC** | | **Number of C** | **GAIIX CE** |
| --- | --- | --- | --- | --- | --- | --- | --- | --- | --- | --- | --- | --- | --- |
| **7941** | 4.8 | 96431662 | 20 | 25589936 | 5 | 2-3 | 14261391 | 153 | 432 | 77 | 433 | | 101 |
| **9701** | 4.7 | 78263916 | 17 | 32560982 | 7 | 6-8 | 6741910 | 73 | 551 | 172 | 550 | | 239 |
| **9717** | 5.2 | 94212559 | 18 | 21988195 | 4 | 2-3 | 5965830 | 58 | 886 | 264 | 892 | | 260 |
| **9432** | 5.0 | 123304239 | 25 | 20095895 | 4 | 2-3 | 13721929 | 141 | 432 | 132 | 438 | | 248 |
| **9443** | 5.1 | 76188990 | 15 | 16402983 | 3 | 2-3 | 4797891 | 48 | 758 | 221 | 760 | | 354 |
| **9806** | 4.2 | 92474422 | 22 | 22473376 | 5 | 2-3 | 5863681 | 71 | 310 | 93 | 310 | | 153 |
| **9807** | 5.1 | 76028093 | 15 | 39028869 | 8 | 6-8 | 12384096 | 124 | 781 | 267 | 782 | | 209 |
| **9808** | 5.0 | 121740160 | 24 | 23471449 | 5 | 6-8 | 6426649 | 66 | 479 | 141 | 479 | | 171 |
| **9809** | 4.9 | 77419476 | 16 | 19929408 | 4 | 6-8 | 9948036 | 103 | 808 | 303 | 809 | | 361 |
| **T1-4** | 4.7 | 84010153 | 17 | 20011788 | 4 | 6-8 | 7371984 | 77 | 448 | 145 | 449 | | 108 |

454 SR number: Number of 454 single reads; Cov. 454 SR: Coverage of the 454 Single Reads (=454SR/Genome size); 454 MPr number: Number of 454 Mate-Paired reads nb; Cov. 454 MP: Coverage of the 454 Mate-Paired reads (=454MPr/Genome size); MP FS: Mate-Paired Fragment Size; GAIIX SR number: Number of Illumina reads; Cov. GAIIX SR: Coverage of the GAIIX Single Reads; Contigs number ≥500nt: Number of contigs with a size length ≥500 nt; Number of SC: Number of scaffolds; Number of C: Number of contigs; GAIIX CE: Number of sequence errors corrected by Illumina single reads.
