# Supplementary material for: A Tribute to Disorder in the Genome of the Bloom-Forming Freshwater Cyanobacterium Microcystis aeruginosa
Source: PLoS One. 2013 Aug 12;8(8):e70747. doi: 10.1371/journal.pone.0070747 (PMC3741299; doi:10.1371/journal.pone.0070747)
Supplement: Table S2 — Distribution of the tRNA genes in the twelve Microcystis aeruginosa genomes (including the two previously-available genomes of PCC 7806 and NIES-843). (DOCX) [file pone.0070747.s002.docx]

**Supplemental Table 2**

| **Strains** | **7941** | **9701** | **9432** | **9443** | **9701** | **9717** | **9806** | **9807** | **9808** | **9809** | **T1-4** | **7806** | **NIES-843** |
| --- | --- | --- | --- | --- | --- | --- | --- | --- | --- | --- | --- | --- | --- |
| Ala tRNA | 3 | 3 | 3 | 3 | 3 | 3 | 3 | 3 | 3 | 3 | 3 | 3 | 3 |
| Arg tRNA | 4 | 4 | 4 | 4 | 4 | 4 | 4 | 4 | 4 | 4 | 4 | 4 | 4 |
| Asn tRNA | 1 | 1 | 1 | 1 | 1 | 1 | 1 | 1 | 1 | 1 | 1 | 1 | 1 |
| Asp tRNA | 1 | 1 | 1 | 1 | 1 | 1 | 1 | 1 | 1 | 1 | 1 | 1 | 1 |
| Cys tRNA | 1 | 1 | 1 | 1 | 1 | 1 | 1 | 1 | 1 | 1 | 1 | 1 | 1 |
| Gln tRNA | 1 | 1 | 1 | 1 | 1 | 1 | 1 | 1 | 1 | 1 | 1 | 1 | 1 |
| Glu tRNA | 1 | 1 | 1 | 1 | 1 | 1 | 1 | 1 | 1 | 1 | 1 | 1 | 1 |
| Gly tRNA | 3 | 3 | 3 | 3 | 3 | 3 | 3 | 3 | 3 | 3 | 3 | 3 | 3 |
| His tRNA | 1 | 1 | 1 | 1 | 1 | 1 | 1 | 1 | 1 | 1 | 1 | 1 | 1 |
| Ile tRNA | 1 | 1 | 1 | 1 | 1 | 1 | 1 | 1 | 1 | 1 | 1 | 1 | 3 |
| Leu tRNA | 5 | 5 | 5 | 5 | 5 | 5 | 5 | 5 | 5 | 5 | 5 | 5 | 5 |
| Lys tRNA | 1 | 1 | 1 | 1 | 1 | 1 | 1 | 1 | 1 | 1 | 1 | 1 | 1 |
| Met tRNA | 3 | 3 | 3 | 3 | 3 | 3 | 3 | 3 | 3 | 3 | 3 | 3 | 2 |
| Phe tRNA | 1 | 1 | 1 | 1 | 1 | 1 | 1 | 1 | 1 | 1 | 1 | 1 | 1 |
| Pro tRNA | 3 | 3 | 3 | 3 | 3 | 3 | 3 | 3 | 3 | 3 | 3 | 3 | 3 |
| Ser tRNA | 4 | 4 | 4 | 4 | 4 | 4 | 4 | 4 | 4 | 4 | 4 | 4 | 4 |
| Thr tRNA | 2 | 3 | 2 | 3 | 3 | 3 | 3 | 3 | 2 | 3 | 3 | 3 | 3 |
| Trp tRNA | 1 | 1 | 1 | 1 | 1 | 1 | 1 | 1 | 1 | 1 | 1 | 1 | 1 |
| Tyr tRNA | 1 | 1 | 1 | 1 | 1 | 1 | 1 | 1 | 1 | 1 | 1 | 1 | 1 |
| Val tRNA | 2 | 2 | 2 | 2 | 2 | 2 | 2 | 2 | 2 | 2 | 2 | 2 | 2 |
| Total | 40 | 41 | 40 | 41 | 41 | 41 | 41 | 41 | 40 | 41 | 41 | 41 | 42 |
