# Supplementary figures and images for: A Tribute to Disorder in the Genome of the Bloom-Forming Freshwater Cyanobacterium Microcystis aeruginosa
Source: PLoS One. 2013 Aug 12;8(8):e70747. doi: 10.1371/journal.pone.0070747 (PMC3741299; doi:10.1371/journal.pone.0070747)

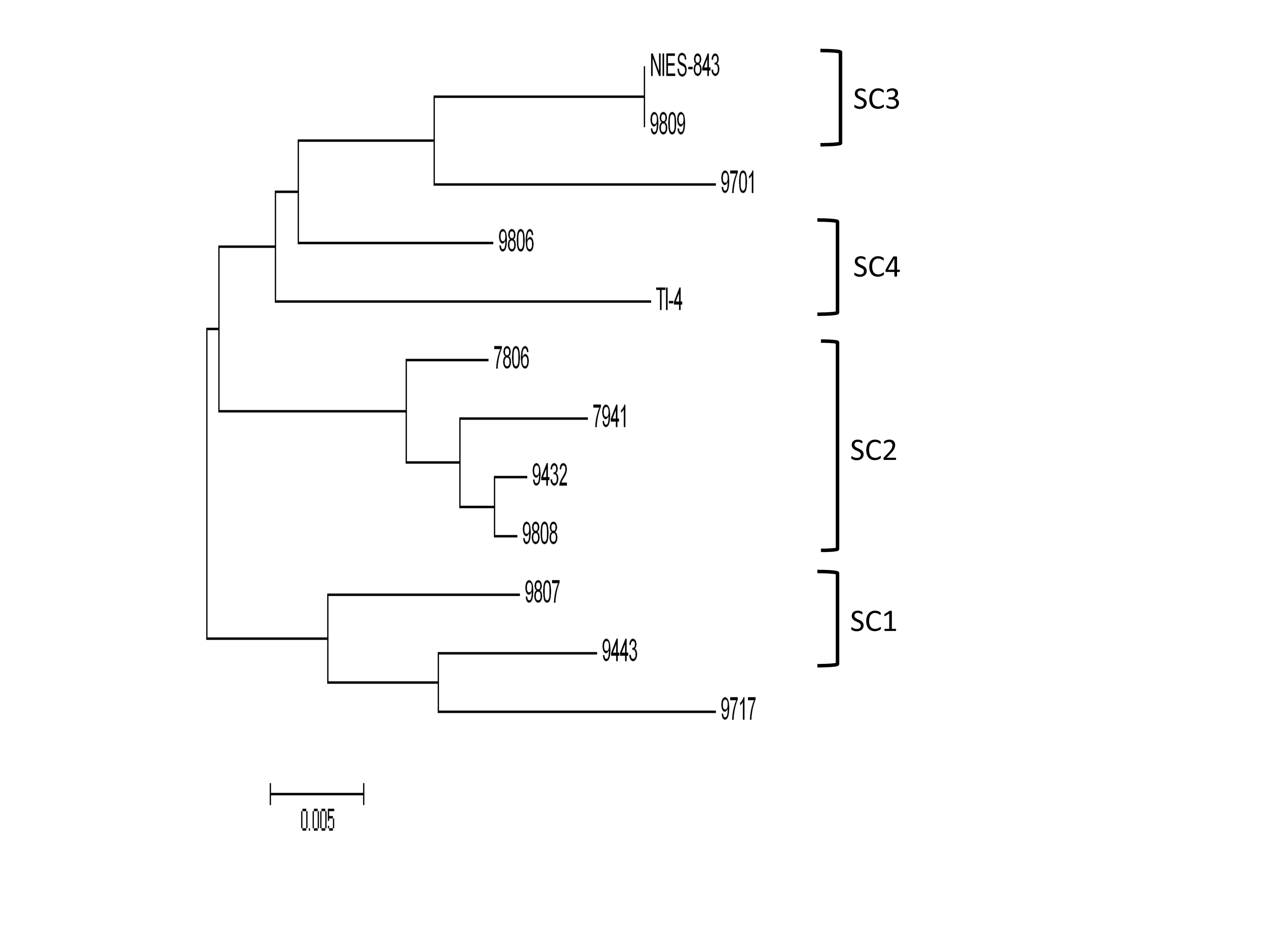

Supplement: Figure S1 — Phylogenetic relationships (Neighbor-joining method) between the twelve Microcystis aeruginosa genomes (including the two previously-available genomes of PCC 7806 and NIES-843) based on the alignment of the 16S–23S rDNA Internal Transcribed Spacer. (TIF) [file pone.0070747.s006.tif]

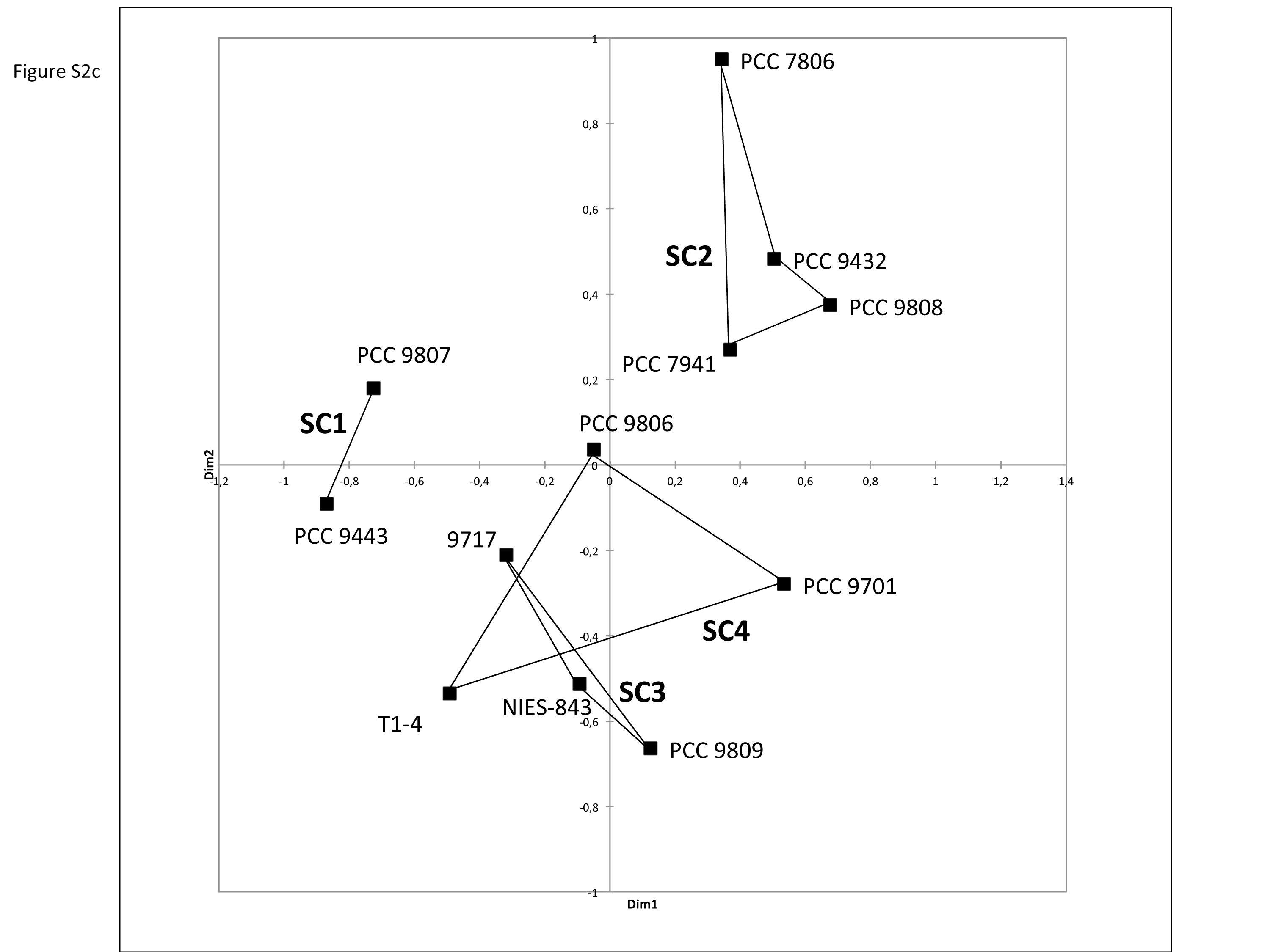

Supplement: Figure S2 — Non-metric MDS analysis performed on the matrix of synteny values estimated between the twelve Microcystis aeruginosa genomes (including the two previously-available genomes of PCC 7806 and NIES-843). SC1, 2, 3 & 4: Subclades have been defined in the phylogenetic tree based on the core genome (see Fig. 3) (TIF) [file pone.0070747.s007.tif]

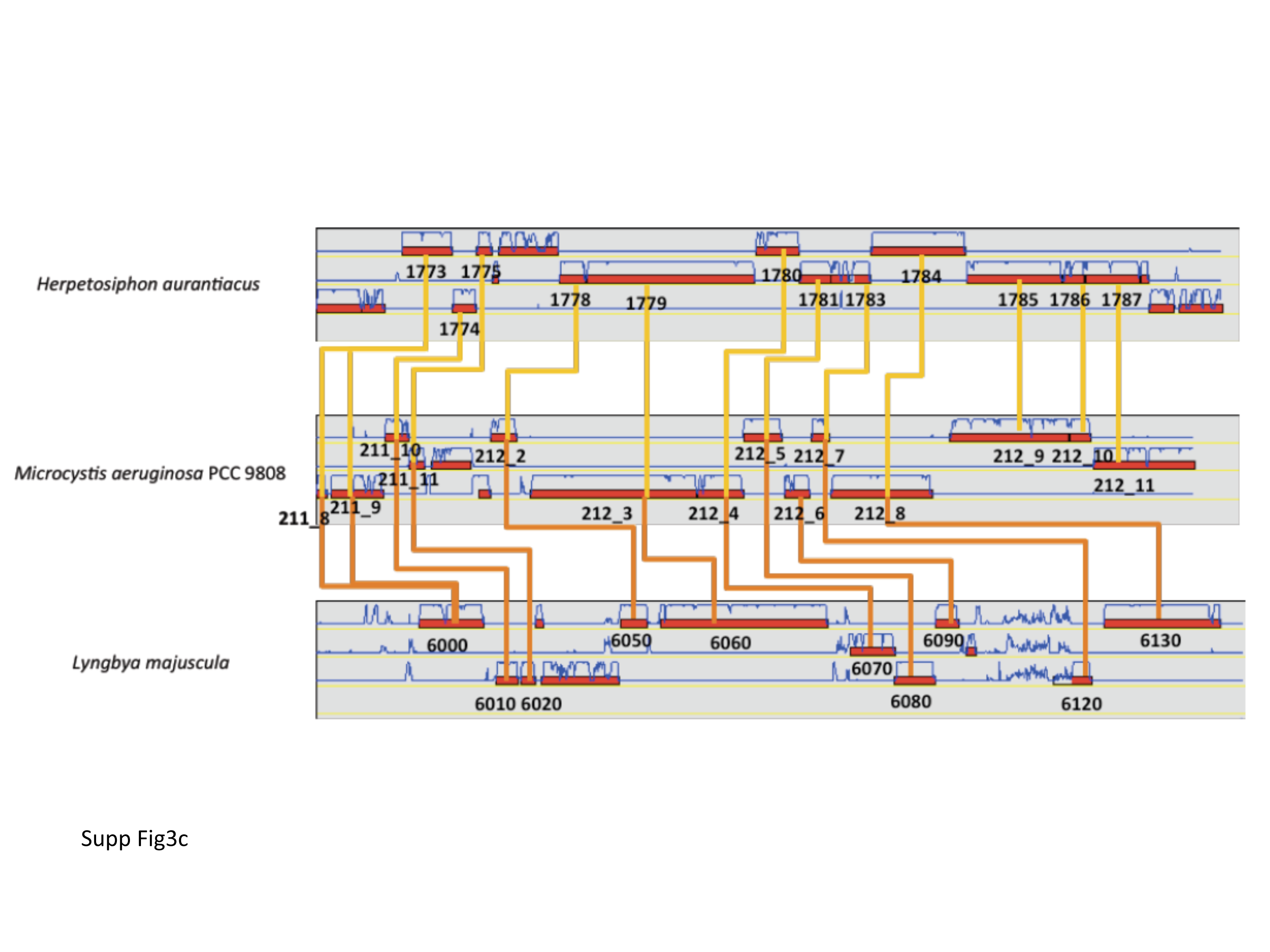

Supplement: Figure S3 — Comparative gene organization of a strain specific gene cluster found in Microcystis aeruginosa PCC 9808 (MICG_2110008- MICG_2120011) with that found in Lyngbya majuscula and Herpetosiphon auriantiacus genomes. (TIF) [file pone.0070747.s008.tif]

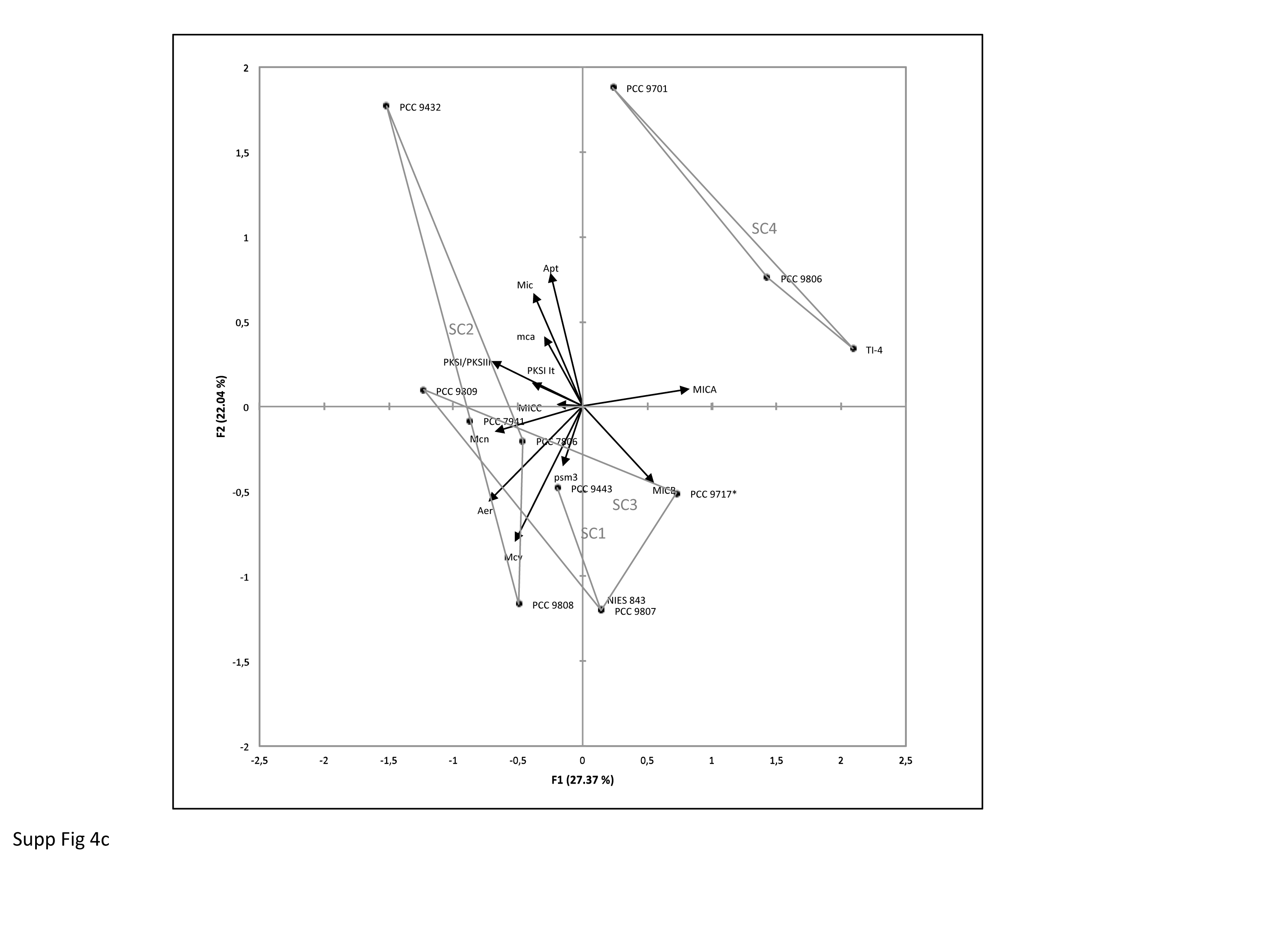

Supplement: Figure S4 — Correspondence analysis performed on the distribution (coded as 1 when all the genes of the cluster were present in a strain; 0.5 when the cluster was not complete and 0 when no gene of the cluster was present) of the clusters of genes involved in the biosynthesis of secondary metabolites among the twelve M. aeruginosa strains (including the two previously-available genomes of PCC 7806 and NIES-843). SC1, 2, 3 & 4: Sub-clades defined in the phylogenetic tree based on the core genome (see Fig. 3). (TIF) [file pone.0070747.s009.tif]
